# Learning performance and GABAergic pathway link to deformed wing virus in the mushroom bodies of naturally infected honey bees

**DOI:** 10.1242/jeb.246766

**Published:** 2024-07-10

**Authors:** Szymon Szymański, David Baracchi, Lauren Dingle, Alan S. Bowman, Fabio Manfredini

**Affiliations:** ^1^ School of Biological Sciences, University of Aberdeen, Zoology Building, Tillydrone Avenue, Aberdeen, AB24 2TZ, UK; ^2^ Department of Biology, University of Florence, Via Madonna del Piano 6, 50019 Sesto Fiorentino, Italy

**Keywords:** *Apis mellifera*, Proboscis extension reflex, Gene expression, DWV, Learning enhancement

## Abstract

Viral infections can be detrimental to the foraging ability of the western honey bee, *Apis mellifera*. The deformed wing virus (DWV) is the most common honey bee virus and has been proposed as a possible cause of learning and memory impairment. However, evidence for this phenomenon so far has come from artificially infected bees, while less is known about the implications of natural infections with the virus. Using the proboscis extension reflex (PER), we uncovered no significant association between a simple associative learning task and natural DWV load. However, when assessed through a reversal associative learning assay, bees with higher DWV load performed better in the reversal learning phase. DWV is able to replicate in the honey bee mushroom bodies, where the GABAergic signalling pathway has an antagonistic effect on associative learning but is crucial for reversal learning. Hence, we assessed the pattern of expression of several GABA-related genes in bees with different learning responses. Intriguingly, mushroom body expression of selected genes was positively correlated with DWV load, but only for bees with good reversal learning performance. We hypothesise that DWV might improve olfactory learning performance by enhancing the GABAergic inhibition of responses to unrewarded stimuli, which is consistent with the behavioural patterns that we observed. However, at higher disease burdens, which might be induced by an artificial infection or by a severe, natural *Varroa* infestation, this DWV-associated increase in GABA signalling could impair associative learning as previously reported by other studies.

## Teaser

**Summary:** Description of a virus-associated increase in learning in honey bees and proposal of a mechanism based on GABA to explain the interplay between infection and cognition in the insect brain.

## INTRODUCTION

Honey bee colonies are susceptible to a wide range of parasites, and infections have been associated with incidences of colony death (Cox-Foster et al., 2007; Oldroyd, 2007; Cepero et al., 2014). While infections can lead to increased mortality, more subtle effects can also be detrimental for the colony. For example, infections with the microsporidian *Nosema ceranae* have been associated with fewer chances for bees to perform foraging duties or to carry pollen (Lach et al., 2015), and the Israeli acute paralysis virus has been shown to induce more frequent outbound flights from the hive by foragers in conjunction with a low-quality pollen provision (Dolezal et al., 2019). Also, it has been proposed that such infection-derived behavioural effects are crucial factors in over-wintering losses and colony death, as they affect the well-being and fitness of the entire colony (Chen et al., 2021a; Decourtye et al., 2003).

Deformed wing virus (DWV) is the most common honey bee virus: infections can be lethal to individual bees as well as to the whole colony (Martin et al., 2015). DWV is strongly associated with the parasitic mite *Varroa destructor*, which acts as an efficient vector of the virus (Martin and Brettell, 2019; Chapman et al., 2023). Severe (overt) infections cause a characteristic wing deformity in newly emerged bees (Martin and Brettell, 2019), alterations of the cuticular profiles (Baracchi et al., 2012) and additional symptoms such as bloated abdomen, ataxia and discolouration (de Miranda and Genersch, 2010). However, more subtle consequences of DWV infections have also been reported, particularly when the virus is not transmitted by *Varroa* or persists in the population through chronic (covert) infections (Wells et al., 2016). Behavioural and cognitive impairments such as deficits in olfactory learning and memory are among the reported symptoms associated with covert infections (Chen et al., 2021a; Iqbal and Mueller, 2007; Tang et al., 2021). Although some effects of DWV are not lethal for the individual bee, they can be detrimental at the colony level, as they can impair the ability of workers to effectively forage, therefore threatening colony fitness and survival.

Several studies examining the effect of DWV on the physiology and cognition of honey bees have used artificial infections. In these studies, the severity of the achieved infections is not always reported and it transpires that different modes of infections (e.g. feeding or injection) can produce contrasting results (Chen et al., 2021a; Iqbal and Mueller, 2007; Tang et al., 2021). For example, Iqbal and Mueller (2007) reported virus-associated deficits in both learning and memory using DWV injections, while Chen et al. (2021a) only reported deficits in long-term memory by feeding bees with DWV. In studies that measure cognitive impairment, the typical paradigm used is a change in the proboscis extension reflex (PER) response rate to conditioned odours. During PER, a bee will extend its proboscis in response to sugar being placed on its antennae. PER is readily associated with odours through classical conditioning; therefore, it is a widely used proxy for insect learning and memory (Giurfa and Sandoz, 2012). The system has also been broadly used to investigate reversal learning by conditioning bees to respond to two odours, and then reversing the reinforcements associated with them – sugar stimulation of the antennae or the lack thereof (Mota and Giurfa, 2010). The link between performance in PER and the immune responses triggered by a parasitic infection has been well characterised (Mallon et al., 2003; Scheiner et al., 2003), including the molecular mechanisms underpinning the learning process (Raccuglia and Mueller, 2013). However, the mechanisms that a virus such as DWV exploits to affect PER conditioning remain unclear.

Some of the proposed mechanisms include alterations in olfactory perception, neuronal signalling and carbohydrate metabolism pathways (Chen et al., 2021a; Iqbal and Mueller, 2007; Pizzorno et al., 2021). One mechanism that has not been explored yet is GABAergic neuronal signalling within the mushroom bodies. Mushroom bodies are important integration centres of the insect brain with a key role in learning and memory (Homberg, 1984; Menzel, 2012), and GABA is known to play a role in the modulation of these functions across many insect species (Dupuis et al., 2010). Experiments conducted in honey bees and *Drosophila melanogaster* fruit flies showed that the concentration of GABA in the mushroom bodies as well as the expression of GABA receptors and GABA synthesis genes have a strong antagonistic effect on insect associative learning (Liu et al., 2007; Liu and Davis, 2008; Raccuglia and Mueller, 2013). In these studies, when GABA-related genes were overexpressed, or GABA concentration within the mushroom bodies was experimentally increased, olfactory learning was either completely extinct or heavily impaired. Furthermore, GABA signalling in the mushroom body calyces of honey bees has been shown to play a role in the regulation of reversal learning, a process whereby a bee must disregard a previously learned association to form a new one (Mota and Giurfa, 2010). When bee foragers were injected with a pharmacological agent inhibiting GABA signalling, they were not able to reverse already learned associations (Boitard et al., 2015). However, in this GABA-inhibited state, formation of new olfactory associations and scent discrimination were unaffected (Devaud et al., 2007; Boitard et al., 2015). Thus, the mushroom body GABAergic pathway emerges as a major modulator of olfactory learning processes in the honey bee (Ganeshina and Menzel, 2001) and appears to act in a complex and often counterintuitive fashion. Intriguingly, DWV has been localised in the honey bee mushroom bodies – where it actively replicates (Shah et al., 2009) – and can induce changes in neuronal signalling (Pizzorno et al., 2021), suggesting a direct interaction between DWV and cognitive functions in this brain region that could be mediated by GABA.

GABA has already been described as a potential mechanism to explain the effects of pesticides on honey bee olfactory learning. Numerous types of pesticides are known to decrease the learning performance of bees in a dose-dependent and mode of exposure-dependent manner (Siviter et al., 2018; Carlesso et al., 2020; Cabirol et al., 2023). For example, early exposure to imidacloprid was associated with differential expression of GABA receptor subunit genes (Chen et al., 2021b), while exposure to thiamethoxam was associated with altered expression of synapsin, involved in the release of GABA in the central nervous system (Tavares et al., 2019). Moreover, nectar-borne GABA can directly affect learning and memory in honey bees (Carlesso et al., 2021) and bumblebees (Calderai et al., 2023).

In this study, we used PER to test the associative and reversal learning abilities of honey bees with naturally occurring DWV infections. We then quantified DWV load in the mushroom body of bees that underwent PER, to investigate whether cognitive impairments correlated with DWV load. Finally, we assessed the expression of genes in the GABA pathways in mushroom bodies of bees that showed different responses in reversal learning tests. Notably, our experiments used honey bees that were naturally infected with DWV, clearly separating our experimental approach from previous research where the virus was introduced via injection or oral administration. By using natural infections, we were able to test the effect of a virus on cognition and GABA-related gene expression in a more ecologically relevant context.

## MATERIALS AND METHODS

### Colonies

Colonies of *Apis mellifera* Linnaeus 1758 used in this study were kept in two locations near Aberdeen, Scotland: Cruickshank Botanical Garden, on the University of Aberdeen Kings College campus (grid reference: NJ936085), and in Newburgh, Aberdeenshire (NJ998260). Cruickshank Botanical Garden colonies regularly receive standard *Varroa* treatments and consequently have low *Varroa* infection rates and low DWV levels. Newburgh colonies receive no *Varroa* treatments and have relatively high *Varroa* and DWV levels (Woodford et al., 2022). Preliminary analyses revealed that there was a significant apiary effect in the distribution of DWV loads in the abdomens of foragers used for the simple associative learning assay (Mann–Whitney *U*; *W*=2806.5, *P*=4.53×10^−5^). A real-time qPCR screening of the most common honey bee viruses in Scotland (DWV, ABPV, CBPV and SBV) revealed that only DWV was present at detectable levels in our colonies (Fig. S1).

### Simple associative learning assay

Two colonies in Cruickshank Botanical Garden and two in Newburgh were used as a source of bees for the simple associative learning trials. Foragers returning from foraging trips were captured between 09:00 h and 10:00 h at the colony entrance and transferred to the lab. Here, they were immobilised on ice for harnessing (Scheiner et al., 2013), fed with 3–5 μl of 30% (w/w) sucrose solution and maintained in the harness for an hour before the start of the assays. Harnessed bees were initially checked for appetitive motivation by touching the antennae with a toothpick soaked in 30% sucrose (w/w in water). If they did not exhibit a PER response, they were discarded from the experiment.

Bees were assessed with the PER absolute conditioning test. They were conditioned to respond to citral (Sigma-Aldrich, St Louis, MO, USA) through a 5-time exposure to the odour with reinforcement by touching the antennae with a 30% (w/w) sugar solution. Citral (5 μl) was pipetted onto a piece of filter paper, which was then inserted into a 20 ml syringe. The bees were exposed to citral by expressing the full volume of air from the syringe around 0.5 cm away from the bees' antennae. A general PER olfactory conditioning protocol followed Mota and Giurfa (2010), with only one scent being used and no reversal performed. Foragers that exhibited a PER response to the first citral exposure were discarded. Foragers were then scored from 0 to 4 according to their responses during conditioning (0=no response during all trials, 4=PER response to 4 trials). Thirty minutes after the conditioning was concluded, foragers were exposed to the same odour as a mid-term memory test.

After behavioural testing, bees were frozen in a −80°C freezer and stored until later processing for molecular work.

### Reversal associative learning assay

For this experiment, one hive in Cruickshank Botanical Garden and one in Newburgh were used – foragers were sampled, harnessed and fed as described above. One hour after harnessing, bees were desensitised to water by touching the antennae with a water-soaked toothpick. Then, the bees were tested for sucrose responsiveness by exposure to six sucrose solutions of increasing concentration (0.1%, 0.3%, 1%, 3%, 10% and 30% w/w) with an inter-stimulus interval (ISI) of 2 min (Carlesso et al., 2020). This test controls for the possible effect of different levels of nutritional stress and appetitive motivation, as foragers returning from foraging trips might be in varying nutritional states. Sugar exposures were separated by water exposures to avoid sensitisation. To account for potential laterality in sucrose responsiveness, both antennae were stimulated (Baracchi et al., 2018). Individuals not responding to sucrose after exposure to a final 50% w/w solution and individuals responding to water after 30 min desensitisation were excluded from the subsequent steps. A sucrose responsiveness score (SRS) was calculated as the number of sucrose concentrations eliciting PER responses in bees.

After the initial tests, bees were fed 15 μl 30% (w/w) sucrose solution and maintained in the harness overnight. The following day, a differential conditioning experiment was conducted in two phases using two stimuli: odorant A (1-hexanol) and odorant B (nonanal). The odour was delivered using a syringe, as described above. In the forward learning phase, A was reinforced positively, using 30% (w/w) sucrose solution placed on the antennae, and B was not reinforced (A=CS+, B=CS−; A+ and B−). An hour after conclusion of the forward learning phase, the reversal learning phase was conducted. In the reversal learning phase, the stimuli and reinforcements were switched, with A not reinforced and B positively reinforced with sucrose (A=CS−, B=CS+; A− and B+). In each phase, bees were exposed to the odorants 5 times per odour (total of 10 trials per phase) at an ISI of 10 min in a pseudorandomised sequence (i.e. at most two CS− or CS+ odours were presented in succession).

Individual animals were divided into learners and non-learners according to a mid-term memory retention test. This test was performed 1 h after the differential conditioning was concluded. Bees that responded with PER to odour B and did not respond to odour A were classified as learners, as this was the last conditioning sequence they were exposed to (A− B+). All other bees were classified as non-learners.

After behavioural experiments were concluded, experimental bees were frozen in a −80°C freezer and stored until later processing for molecular work.

### Analysis of data from learning assays

All statistical analyses were conducted in R, version 4.2.0 (http://www.R-project.org/). Data were visualised using ggplot2 and ggpubr libraries. A standard significance threshold of 0.05 was adopted for all analyses.

PER scores in the simple associative learning assay were analysed using linear regression with the base R functions lm() and aov(). Tests were performed between PER score and log_10_-transformed mushroom body DWV load, PER score and log_10_-transformed abdomen DWV load, and also between mushroom body and abdomen log_10_-transformed DWV load. Test assumptions were checked by examining residuals-fits plots and Anderson–Darling tests on residuals [ad.test() from nortest library]. Differences in DWV load according to performance in the mid-term memory test were assessed using a Wilcoxon rank test.

Bees' learning performances in the reversal learning assay were analysed with a generalised linear mixed model (GLMM) (Baracchi et al., 2020). The GLMM was designed with a binomial error structure–logit-link function (glmer function in package lme4). The iterative algorithm BOBYQA was used to optimise the models. We retained the significant model with the highest explanatory power.

The selected model for the reversal learning phase included ‘bee response’ (1=PER, 0=no PER) as the dependent variable, ‘CS’ and ‘group’ (i.e. learners or non-learners) as fixed factors, and ‘DWVlog’ and ‘conditioning trial’ as covariates. In all models, the individual identity ‘ID’ was entered as a random factor to account for repeated measures. The difference in DWV load between learners and non-learners was assessed using a Mann–Whitney test.

### Quantification of DWV load using RT-qPCR

We used a molecular assay to quantify viral load in different tissues from honey bee samples collected after the simple associative learning assay (mushroom bodies and whole abdomens of 60 bees, abdomens only for 126) and reversal associative learning assay (mushroom bodies alone of 203 bees). Mushroom bodies were dissected on dry ice following Veiner et al. (2022). In short, the head was separated from the abdomen, antennae and proboscis. The cuticle between the compound eyes was scraped off, the hypopharyngeal glands and the ocelli were carefully removed, and the mushroom bodies were separated from the rest of the brain with two symmetrical cuts between the mushroom bodies and the optical lobes (Sen Sarma et al., 2009). The central complex on the ventral side of the mushroom bodies was removed before further processing. Mushroom bodies were then homogenised in a Tissue Lyser II device (Qiagen, Hilden, Germany) using 1 ml TRIzol and 2.3 mm zirconia beads (Thistle Scientific, Glasgow, UK). Total RNA was isolated following a routine TRIzol protocol and the final elution was performed in 20 µl of nuclease-free water. Whole abdomens were processed in the same way, except for the final elution, which was performed in 50 µl of nuclease-free water. To quantify viral load, 300 ng of RNA was reverse transcribed into cDNA using the iScript^TM^ cDNA Synthesis Kit (BioRad Laboratories Inc., Hercules, CA, USA), following the manufacturer’s protocol. cDNA samples were used as templates to run qPCR reactions with three sets of primers: specific for strain A of the virus (DWV-A), specific for strain B (DWV-B) and global primers capable of detecting both strains (PAN primers). Primer sequences, qPCR conditions and viral load estimation methods were the same as described in Bradford et al. (2017). Throughout this work, DWV load will be expressed in genome equivalents.

Preliminary analyses revealed that there was a strong correlation between DWV-A and DWV-B load and overall DWV load as detected with PAN primers, in both the abdomen and mushroom body samples (Fig. S2, Table S2). Therefore, we opted to use only DWV load as detected with PAN primers for this study.

### Sample selection for GABA gene expression assay

**Fig. 1.**
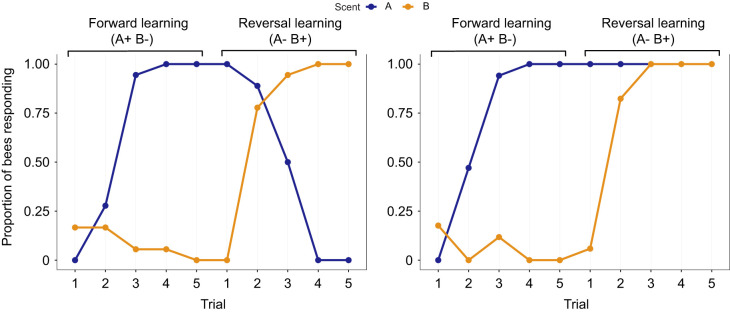
**Response graphs showing the proportion of bees responding with the proboscis extension reflex (PER) to odours A (1-hexanol) and B (nonanal).** Subsets of bees represented in the figure were used for further gene expression analysis using RT-qPCR. The experiment was conducted in two phases, switching the reinforcement associated with odorants between the phases (A+, B−→A−, B+). Left: responses of bees belonging to the GR group (good reversers: good learning ability and efficient reversal). Right: responses of the BR group (bad reversers: good learning ability but not efficient reversal). *N*=18 for both groups.

The learner group described above was subsampled to represent bees that had efficiently formed and reversed olfactory associations (Fig. 1): this subsample will be referred to as good reversers (GR) for simplicity. Similarly, the non-learner group was subsampled to obtain bees that efficiently formed olfactory associations but did not reverse them (Fig. 1), which will be referred to as bad reversers (BR). The selection was made based on bees' ability to pass or fail three behavioural criteria (details are shown in Table 1). This selection process resulted in a large number of samples being allocated to the BR group and only 18 samples to the GR group. Therefore, BR samples were pseudorandomly selected to also provide a matched sample size of 18.

**Table 1.**
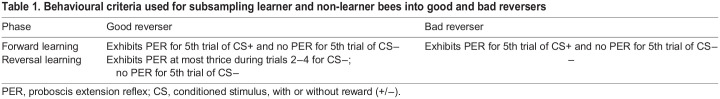
Behavioural criteria used for subsampling learner and non-learner bees into good and bad reversers

### Preparation of RNA samples for gene expression analysis

Total RNA extracted from the mushroom bodies of the GR and BR bees (as described above) was diluted with nuclease-free water to a final volume of 40 µl. As residual genomic DNA can introduce bias to gene expression assays, it was digested using DNase-I (Zymo Research Corp, Irvine, CA, USA) according to the manufacturer’s protocol. After incubation at 23–25°C for 15 min, the RNA was purified and concentrated using the Zymo Research RNA Clean and Concentrator Kit following the manufacturer’s protocol. The only exception to this was the final RNA elution volume, which was performed in 25 µl of nuclease-free water. Afterwards, samples were analysed with a NanoDrop^TM^ One spectrophotometer (ThermoFisher Scientific, Waltham, MA, USA) to determine purity and yield.

To synthesise cDNA, aliquots constituting 250 ng of RNA were diluted with nuclease-free water to a final volume of 15 µl and processed using the iScript^TM^ cDNA Synthesis Kit (BioRad Laboratories Inc.) as per the manufacturer’s protocol. Three additional RNA aliquots were processed without reverse transcriptase to provide non-enzyme controls (NECs).

### Expression analysis of honey bee GABA genes with qPCR

The selected GABA-related genes (Table 2) were *Rdl* (ionotropic GABA-A receptor), *Camkii* (calmodulin-dependent protein kinase II), *Syn* (synapsin), *Abat* (4-aminobutyrate transferase) and *Gad1* (glutamate decarboxylase). Housekeeping genes used for normalisation were *RpS18* (ribosomal protein S18) and *RpL32* (ribosomal protein L32): these were sourced from Deng et al. (2020). A full description of the gene selection process as well as primer design and validation can be found in the Supplementary Materials and Methods. The complete candidate gene list is deposited in Table S1.

**Table 2.**
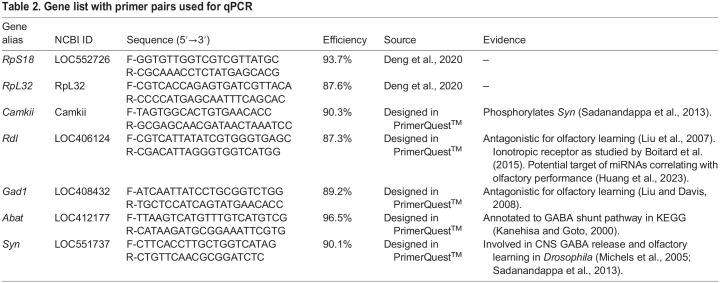
Gene list with primer pairs used for qPCR

A total of 34 samples (GR and BR combined) were assayed in duplicate, with each gene tested on a separate 96-well plate for all samples, including three NECs and three water controls. Assays were performed on a BioRad C1000 Touch^TM^ Thermal Cycler with the CFX96^TM^ Optical Reaction Module attachment. Plates were pre-incubated at 95°C for 3 min, followed by 40 amplification cycles (95°C for 3 s, 60°C for 20 s, 72°C for 3 s), finishing with a 4 min elongation at 72°C. Data from the thermocycler were imported into BioRad CFX Manager software to examine melting curves; thereafter, quantification cycle (*C*_q_) values were exported for further data processing and statistical analyses with R. Sample duplicates were averaged and the *C*_q_ data were normalised using a modified Pfaffl method, which allows for the use of two reference genes (Hellemans et al., 2007; Vandesompele et al., 2002).

Prior to the analysis, all data for one sample in the BR group and one sample in the GR group were removed because of extreme expression values across all genes. The high expression values were consistent for GABA-related genes and the housekeeping genes. Thus, we concluded that they were likely to be a result of biased processing rather than biological variation. For *Gad1* data, an additional GR sample had to be removed because of a lack of replication in the qPCR assay. Differences in GABA-related gene expression between GR and BR groups were tested using separate two-sample *t*-tests for each gene, as the assumptions for nested ANOVA could not be met. This was achieved using the base function t.test(). Repeated testing was accounted for using a Bonferroni correction (VanderWeele and Mathur, 2019). The assessment of *t*-test assumptions was performed using ad.test() and levene_test() functions from nortest and rstatix libraries, respectively.

Changes in gene expression according to DWV load were assessed using a generalised linear model (GLM) with the R function glm(). The explanatory variables were ‘MB log_10_ DWV count’, ‘behavioural group’ (GR or BR), ‘gene’, as well as interaction terms between ‘MB DWV log_10_×behavioural group’, and ‘MB DWV log_10_×gene’. A gamma log link function was used for this analysis because expression data are continuous and non-negative. Validity of the model was checked with an analysis of deviance using the base function pchisq(), by subtracting the model deviance from null deviance and testing the value against a χ^2^ distribution for the model's degrees of freedom. The model was able to explain a significant proportion of the data deviance (χ^2^=23.15, d.f.=11, *P*=0.017). A null model was also a worse fit for the data as demonstrated by the Akaike information criterion (AIC) value (482.62 for the model presented; 491.60 for the null model).

## RESULTS

### Performance in simple learning does not correlate with viral load

**Fig. 2.**
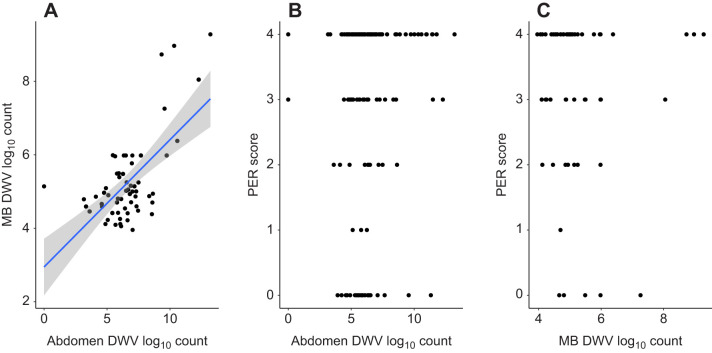
**Regression analyses between PER score and deformed wing virus (DWV) load data in a simple associative learning test.** Scatterplots of mushroom body (MB) DWV count against abdomen DWV count (A), PER score against mushroom body DWV load (B) and PER score against abdomen DWV load (C). A total of 186 bees were assessed using the associative learning assay. Neither abdomen nor mushroom body DWV load was significantly associated with performance (PER score) in this assay. Only mushroom body and abdomen DWV count were significantly associated according to linear regression analysis (*F*_1,58_=39,41, *P*=4.73×10^−8^). Line of fit is *y*=2.985+0.346*x*; shading represents 95% confidence intervals around the fit. *N*=60 for A and C; *N*=186 for B.

Linear regression analyses showed that neither mushroom body nor abdomen DWV load explained a significant proportion of variation in PER score data derived from simple associative learning (Table 3). Furthermore, the performance of bees in the mid-term memory test was not significantly associated with DWV load (Wilcoxon rank test; *W*=226, *P*=0.648). However, mushroom body load was significantly associated with abdomen DWV load (Fig. 2). Both the slope and the intercept of this fit were significantly different from zero (one sample *t*-test; slope *t*=6.28, *P*=4.73×10^−8^; intercept *t*=7.67, *P*=2.17×10^−10^). Regression assumptions were met, with small departures from normality, which were deemed not detrimental to the analysis. This supports the notion that mushroom body DWV loads are representative of overall disease burden. Thus, mushroom body DWV loads were consistently used for the following experiments. There was a significant effect of apiary on PER scores for this set of tests (Mann–Whitney *U*-test; *W*=31,393, *P*=5.82×10^−6^).

**Table 3.**
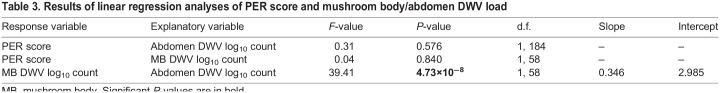
Results of linear regression analyses of PER score and mushroom body/abdomen DWV load

### Performance in reversal learning positively correlates with viral load

**Fig. 3.**
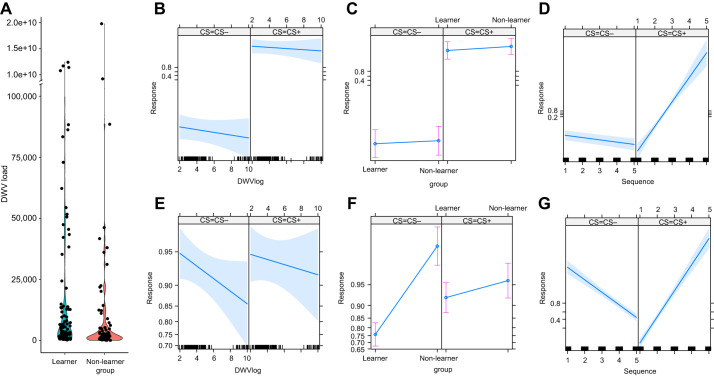
**Correlation between reversal learning and DWV load in honey bee mushroom bodies.** A violin plot presenting DWV load between behavioural groups (A), and effect plots for generalised linear mixed model (GLMM) used to analyse behavioural responses during the reversal learning assay (B–G). A total of 203 bees were processed in the assay (*N*=114 learners; *N*=89 non-learners). B–D present effects during the forward learning phase (odour A=CS+, odour B=CS−) of the experiment and E–G present effects during the reversal phase (odour A=CS−, odour B=CS+). (A) The difference in DWV load between bees that passed the memory test (learners) and bees that did not pass the memory test (non-learners) was significant (Mann–Whitney; *W*=1078, *P*=0.007; please note that the *y*-axis is discontinuous to highlight this significant result). (B) Effect of DWV load on response to odour A (CS+) and odour B (CS−). (C) Effect of behavioural group on response to odour A (CS+) and odour B (CS−). (D) Effect of sequence (trial number) on response to odour A (CS+) and odour B (CS−). (E) Effect of DWV load on response to odour A (CS−) and odour B (CS+). (F) Effect of behavioural group on response to odour A (CS−) and odour B (CS+). (G) Effect of sequence (trial number) load on response to odour A (CS−) and odour B (CS+).

In the forward learning phase of the experiment (A+, B−), bees increased their PER response to the odours throughout the conditioning trials (GLMM, sequence; χ^2^=26.34, d.f.=1, *P*=2.86×10^−7^) and, at the same time, discriminated the CS+ and CS− stimuli (GLMM, CS; χ^2^=23.70, d.f.=1, *P*=1.12×10^−6^; Fig. 3D). DWV load did not affect forward learning (GLMM, MB DWV log_10_; χ^2^=1.43, d.f.=1, *P*=0.23; Fig. 3B).

In the reversal learning phase of the experiment (A−, B+), bees updated their information: they increased their PER response to B+ and decreased their response to A− (GLMM, sequence×CS; χ^2^=293.43, d.f.=1, *P*=2.2×10^−16^; Fig. 3G). However, while the response to stimulus B+ was not influenced by DWV load (Tukey *post hoc* test, *P*=0.17), the response to stimulus A− was negatively associated with increasing DWV load (GLMM, MB DWV log_10_×CS; χ^2^=12.67, d.f.=1, *P*=0.0004; *post hoc* test *P*<0.0001; Fig. 3E). Similarly, learners and non-learners responded equally well to B+ but not to A−, as non-learners failed to stop responding to the unrewarded stimulus over the reversal training (GLMM, Group×CS; χ^2^=47.36, d.f.=1; *P*=5.88×10^−12^; Fig. 3F).

By contrast, DWV load differed between learners and non-learners (Mann–Whitney *U*-test; *W*=1078, *P*=0.007; Fig. 3A). Specifically, learners had higher DWV load in mushroom bodies than non-learners. SRS did not differ between learners and non-learners (Mann–Whitney *U*-test; *W*=4592, *P*=0.224). Also, in this case, SRS did not correlate with DWV load (Spearman test; *r*=−0.06, *P*=0.41). Thus, a possible effect of nutritional stress and appetitive motivation in these foragers seemed negligible.

### Expression of GABA-related genes does not differ between behavioural groups

**Fig. 4.**
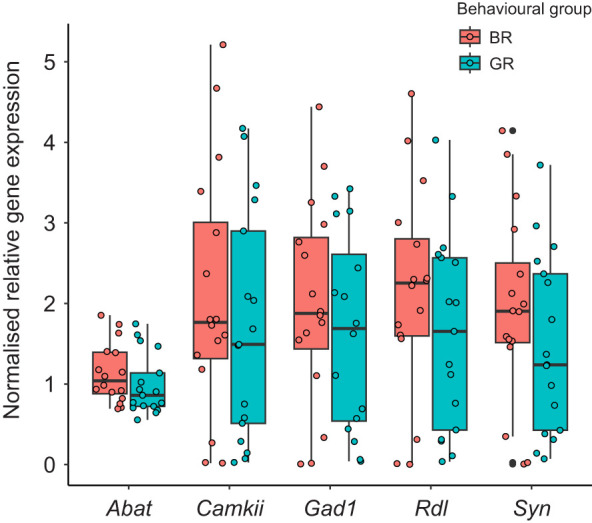
**Relative expression of GABA-related genes in the mushroom bodies of bees exposed to reversal learning.** Boxplots show median and quartile boundaries; whiskers indicate 1.5× interquartile range. Gene expression was assessed for a total of 34 bees subjected to a reversal learning assay, representing subsamples of the bee groups shown in Fig. 3 and selected according to the criteria shown in Table 1. Data points represent expression in each sample. No significant differences were detected between behavioural groups for any gene (two-sample, two-tailed *t*-test); Bonferroni adjustment was used to account for multiple comparisons. *N*=16 for BR for all genes; *N*=16 for GR for *Abat*, *Camkii*, *Rdl*, *Syn*; *N*=15 for GR for *Gad1*.

All groups of gene expression data were normally distributed, except for *Abat* expression in the GR group (Anderson–Darling test; *A*=0.975, *P*=0.010; all other tests *P*>0.15). Equality of variances was also satisfied (Levene's test, all tests *P*>0.80). Therefore, *t*-tests were deemed a valid analysis and a small departure from normality should not be detrimental to it. None of the intra-gene group comparisons proved significant (Table 4); thus, the expression of GABA-related genes in the mushroom bodies did not differ between good and bad reversers. Overall, expression data for *Abat* had lower dispersal than the other genes presented (Fig. 4). A common pattern across all genes was a higher expression in the BR group; however, this trend was not significant.

**Table 4.**
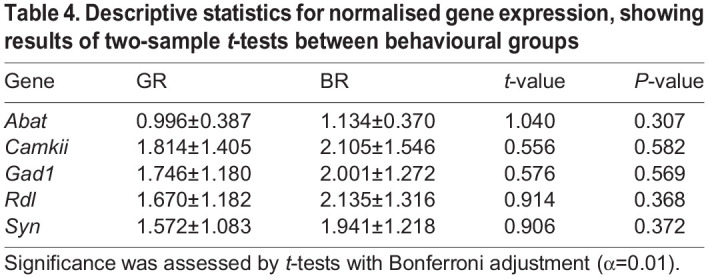
Descriptive statistics for normalised gene expression, showing results of two-sample *t*-tests between behavioural groups

### Expression of GABA-related genes positively correlates with viral load

**Fig. 5.**
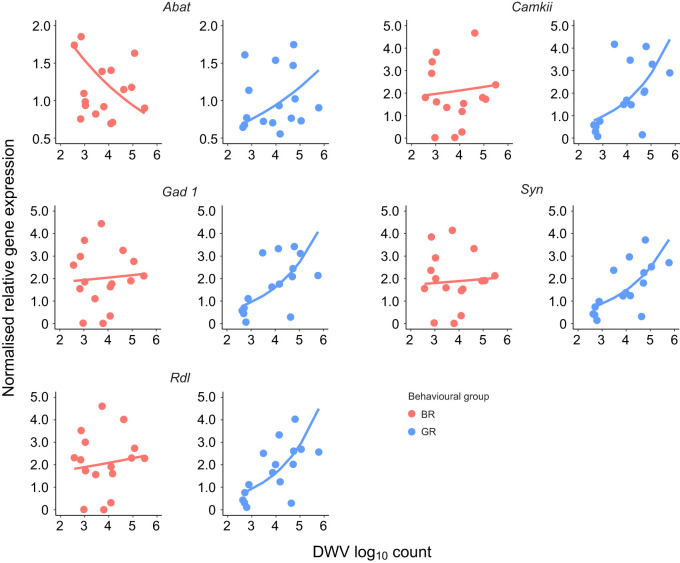
**Correlation between GABA gene expression and DWV load in honey bee mushroom bodies.** Scatterplots show normalised relative expression of five genes related to GABA in the mushroom bodies of bee foragers (*y*-axes), and their mushroom body DWV load (*x*-axes). Gene expression was assessed for a total of 34 bees subjected to a reversal learning assay, representing a subsample of the bee groups shown in Fig. 3 and selected according to the criteria shown in Table 1. The lines shown are fitted values produced by a multivariate, log-linked generalised linear model (GLM). Significant differences were found in slopes of fitted values (β est.=0.473, s.e.=0.108; *t*=4.365, *P*=2.28×10^−5^) between GR and BR data. *N*=16 for BR for all genes; *N*=16 for GR for *Abat*, *Camkii*, *Rdl*, *Syn*; *N*=15 for GR for *Gad1*. Please note *Abat* is on a different *y*-axis scale for visualisation purposes.

The effect of DWV load on GABA-related gene expression was highly significant in GR bees (GLM, MB DWV log_10_; β est.=0.473, s.e.=0.108; *t*=4.365, *P*=2.28×10^−5^) while this was not the case for BR bees (GLM, DWVlog_10_; β est.=−0.248, s.e.=0.134; *t*=−1.849, *P*=0.067). Overall, among GR bees, the expression of all GABA-related genes seemed to increase with increasing log_10_ DWV load (Fig. 5). In the model, expression of the gene *Abat* was used as a baseline and the effect of DWV load on expression was significantly different only for *Rdl* as compared with that baseline (GLM, MB DWV log_10_; β est.=0.343, s.e.=0.170; *t*=2.017, *P*=0.046). However, comparisons of the effect between expression of *Abat* and other genes achieved similar β coefficient estimates and were close to significance (coefficients are given in Table S3).

## DISCUSSION

Here, we combined a set of simple and reversal associative learning tests to characterise the cognitive performance of honey bee foragers naturally infected with DWV and spanning a broad range of viral loads. We then investigated the expression levels of a selected pool of GABA-related genes to assess the effect of chronic infection with DWV on these key regulators of neural function. The performance of bees in the simple associative learning test did not significantly vary in response to DWV: this held true for viral load in both mushroom bodies and abdomens. Mushroom body DWV load was significantly predicted by abdomen DWV load, which deems our use of mushroom body load throughout this work representative of overall load. In the reversal learning assay instead, learners had significantly higher DWV load than non-learners. In an analysis of behavioural responses, bees with a higher DWV load had lower responsiveness to CS− in the reversal phase, thus enhancing their learning performance by inhibiting the response to an unrewarded stimulus. Good and bad reversers did not differ in expression levels for the tested GABA-related genes. However, the expression of those genes increased with DWV load in good reversers.

The fact that PER learning score was not associated with either mushroom body or abdomen DWV load may be explained as follows: DWV does not impair simple associative learning. Such a finding is in direct opposition to previous work, where DWV infection induced learning deficits in a similar assay (Iqbal and Mueller, 2007). This discrepancy may be explained by some key differences in the methods that the two studies adopted, as Iqbal and Mueller (2007) artificially infected adult bees using injections of DWV, while our study assessed bees that were naturally infected. This is a key difference from several points of view: the modality of transmission of a viral parasite (i.e. artificial injection versus natural infection) can dictate how the virus behaves in the host (e.g. viral replication) and can also affect how the host responds (Gisder et al., 2018), as piercing the cuticle with a syringe to deliver the lysate very likely triggers a different chain of reactions than acquiring the virus naturally through parasitisation by *Varroa* or contact with infected nestmates. Unfortunately, in our study it was not possible to establish the source of the natural infections that we quantified, whether it was oral transmission between adult bees, oral transmission between adult bees and larvae, transmission when a larva was parasitised by a mite, or transmission when a pupa was parasitised by a mite. Furthermore, it is impossible to assess whether the DWV levels that we measured were the result of a single or multiple infection events, the latter being more likely in a crowded bee colony. We know that all these factors might play an important role in how the infection dynamics affect the host. In terms of the viable viral load in the two studies, a comparison is impossible as Iqbal and Mueller (2007) do not report viral load in infected bees or DWV lysate used for their experiments. Interestingly, experiments using artificial DWV infections such as Iqbal and Mueller (2007) can achieve markedly higher DWV load than natural infections. For example, one study reported an average of 10 DWV log_10_ genome equivalents in artificially infected bees (Pizzorno et al., 2021), which is on a par with the most severe natural infections observed in our bee colonies. Although Pizzorno et al. (2021) quantified their viral load differently to us, it seems plausible that infection levels in Iqbal and Mueller (2007) were overall significantly higher than observed in our study, and a DWV-driven learning deficit might be specific to such extremely severe, acute infections. This is partially supported by another study (Chen et al., 2021a), where bees were fed DWV lysate and infection levels in the brain reached a DWV copy number of 10^5^–10^6^ at 100 h post-infection (thus comparable to our study). There, the conditioning responses of control and infected bees were strikingly similar: they only differed in long-term memory (Chen et al., 2021a). Furthermore, it has already been demonstrated that some symptoms of DWV infection, such as wing asymmetry, develop in a viral load-dependent manner (Brettel et al., 2017), lending additional support to this hypothesis.

In contrast with the simple associative learning assay, viral load affected the performance of foragers in the reversal learning assay. Foragers with higher infection levels were more likely to show inhibition of responses when an odour stimulus was no longer associated with a food reward (i.e. bees with higher DWV load responded less to previously rewarding stimuli). Such a response seemingly provides a fitness benefit, as responses to negative stimuli are needless as no reward is obtained. Thus, the enhanced inhibition we observed can be referred to as an enhancement in learning performance associated with DWV, because highly infected individuals responded more appropriately to the stimuli they were presented with. Such a result also explains why no effect on performance was seen in the simple associative assay. Basically, there was no negative stimulus in the associative learning assay, as one odour was used, and it was rewarded throughout the assay. Thus, if DWV-associated learning enhancement is driven by inhibition of responses to negative stimuli, the effect simply could not be observed in the associative learning assay.

Expression of GABA-related genes did not differ between good and bad reversers. This is in opposition to what we initially hypothesised, as previous research has shown that activation of ionotropic GABA receptors is necessary for successful reversal learning (Boitard et al., 2015). Although there are other signalling pathways and parts of the brain – not assessed in our study – that are responsible for learning modulation (Müller, 2012), GABA signalling in the mushroom bodies has been strongly linked to the regulation of reversal learning. Specifically, the experimental inhibition of mushroom body GABAergic signalling was shown to completely stop reversal learning in honey bees (Devaud et al., 2007). Our selection of candidate genes includes not only those encoding ionotropic receptors (*Rdl*) but also genes involved with the synthesis (Liu and Davis, 2008) and release of GABA (Liu et al., 2007), and therefore these results do not seem to support a link between expression of GABA-related genes and an individual's ability or inability to perform reversal learning. One aspect worthy of future investigation would be to test whether the facilitation of reversal learning by GABA responds to a threshold effect, so that a minimum degree of GABAergic signalling is necessary for reversal learning, and once that level is reached, learning continues to happen for a certain period. In this scenario, the expression levels of GABA-related genes at the specific time point that we assessed might not reflect the overall activation status of GABAergic pathways, and a more comprehensive time series of observations would be required to explore whether there is any support for this hypothesis.

A key result of our study was the positive correlation between expression of the selected GABA-related genes and DWV load in the mushroom bodies. This was observed for all investigated genes but, intriguingly, it held true only for good reverser bees. Potentially, such an increased GABA-related gene expression could allow a more efficient inhibition of responses to a previously rewarded stimulus. This is supported by the behavioural results of this assay, as learner bees (good reversers are a subsample of these) expressed a negative association between DWV load and responsiveness to CS− in the reversal phase of the reversal associative learning assay. Because the good and bad reversers that were tested for gene expression are subsamples of good and bad learners tested for reversal learning in the PER assay, these considerations can be applied to all bees that underwent the behavioural tests. Furthermore, such an interpretation is congruent with the biological functions of GABA in the mushroom body during the learning process. As outlined above, it is known that mushroom body GABA overwhelmingly inhibits learning in insects. It was experimentally demonstrated that an increased concentration of GABA, or expression of GABA receptors and synthesis genes within the mushroom bodies, is able to stop the learning processes (Liu et al., 2007; Liu and Davis, 2008; Raccuglia and Mueller, 2013). Yet, concurrently, GABA signalling is crucial for successful reversal learning (Boitard et al., 2015), a process that notably involves the inhibition of the response to a previously established association. Although the exact role of GABA signalling in reversal learning performance is not known, it is plausible that it inhibits the responses to already known stimuli, which is consistent with its overall learning-antagonistic action. Such a scenario also reconciles our behavioural and gene expression results. The DWV-associated increase in the expression levels of GABA-related genes could promote the response inhibition observed in infected bee foragers, which in our case was in fact positively correlated with viral load.

Our study disentangles a small portion of the complex interaction between viral infections, learning and gene expression in the honey bee mushroom bodies. At the same time, the results presented here highlight some possible limitations to this work. First, whole mushroom body samples might not provide the appropriate anatomical resolution to demonstrate GABAergic differences in gene expression between bees with different reversal learning abilities. For example, GABAergic control of reversal learning has been localised to the calyxes of the mushroom body (Boitard et al., 2015), while we used whole mushroom bodies. Although our decision to focus on the mushroom bodies was driven by functional evidence, it is important to recognise that the antennal lobes also play a role in the modulation of olfactory learning, both in terms of GABA signalling and in terms of other molecular pathways that could be involved (Müller, 2012; Raccuglia and Mueller, 2013), thus inviting future investigation of this additional brain region. Furthermore, the anterior paired lateral neuron is a GABAergic neuron that projects from the antennal lobe into the mushroom body. This neuron is known to form a memory trace during associative learning and the manipulation of *Gad1* (GABA synthesis enzyme) expression in the neuron modulates learning ability in *Drosophila* (Liu and Davis, 2008). Expression patterns of *Rdl* (ionotropic GABA_A_ receptor) in the mushroom body closely follow the projections of that neuron (Liu et al., 2007). Therefore, the GABAergic mediation of olfactory reversal learning may occur at different levels. Future works could thus utilise methods with a higher resolution, such as single-cell sequencing to distinguish between the different neuron types in and around the mushroom body (Zhang et al., 2022). An additional limitation, already highlighted in this discussion, pertains to the focus on a single time point for the screening of GABA-related gene expression. As evidence shows that GABA might operate in a highly time-dependent fashion (Raccuglia and Mueller, 2013), a wider range of sampling time points should be tested in the future to provide a more comprehensive characterisation of the expression patterns of GABA-related genes. Finally, another limitation of our study can be seen in the use of colonies from two different apiaries, which might have been exposed to different stressors: above all, the application of anti-*Varroa* treatments in Cruickshank Botanical Garden colonies but not in Newburgh colonies. The focus on the two apiaries was a key element in our experimental design, as we wanted to obtain foragers with a broad range of DWV infections (high in Newburgh and low or absent in Cruickshank Botanical Garden colonies) and the miticide was applied at the end of the foraging season, after all our analyses were completed. We cannot exclude, however, any possible chronic effect of anti-*Varroa* treatments on two of the colonies that we used. Even though limited long-lasting effects are known (Colin et al., 2021), the impact of miticides on the cognitive ability of foragers has not yet been fully characterised.

A more complex picture of how different effects of DWV interact must also be considered. For instance, recent transcriptomic evidence suggests that DWV infections induce accelerated behavioural maturation (Traniello et al., 2020). Thus, more heavily infected foragers could be younger than non-infected foragers, and therefore have less foraging experience; alternatively, they could show precocious signs of age-related cognitive decline. In either case, the performance in a PER learning paradigm could be affected more by the behavioural maturation than by the virus per se. Although PER performance of honey bees has been shown to more strongly associate with caste rather than forager age (Ray and Ferneyhough, 1999), knowing the age of infected foragers in future experiments will surely help to disentangle the role played by different DWV-associated symptoms. Furthermore, it is known that DWV infections can alter foraging patterns in honey bees and by doing so they might induce nutritional stress (Benaets et al., 2017), but we have some evidence that this might not be the case in our experimental bees. Firstly, the sucrose responsiveness test that we ran at the beginning of the reversal learning paradigm did not reveal any significant difference in the levels of hunger of foragers with high DWV load. Secondly, the expression of a suite of genes that play a key role in the glycolysis pathway did not differ between foragers with high versus low DWV load, when assessed in the mushroom bodies of a separate cohort of bees (F.M., unpublished data). We cannot exclude, however, that harbouring chronic infection since pre-imaginal stages might have implications for the regulation of the GABA pathway that are long lasting.

Our study represents a first step in the understanding of the effect of natural infections on honey bee cognitive abilities and highlights significant differences from what was previously reported for artificial infections. This testifies to the importance of expanding the assessment to a range of modalities and intensities of infections, which will provide new insights into this fascinating host–parasite system and a better understanding of its relevance in an ecological context.

## Supplementary Material

10.1242/jexbio.246766_sup1 Supplementary information
