# Supplementary material for: Learning performance and GABAergic pathway link to deformed wing virus in the mushroom bodies of naturally infected honey bees
Source: J Exp Biol. 2024 Jul 10;227(13):jeb246766. doi: 10.1242/jeb.246766 (PMC11418184; doi:10.1242/jeb.246766)
Supplement: Supplementary information [file jexbio-227-246766-s1.pdf]

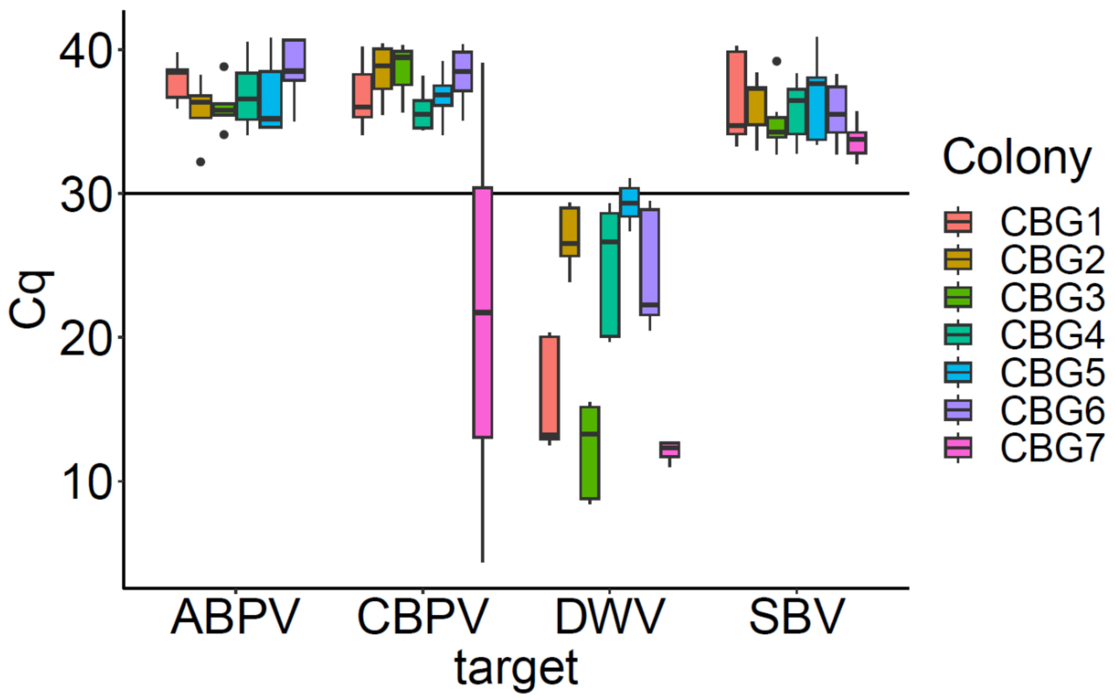

Fig. S1. Boxplots showing Cq in a qPCR-based viral assay at 7 colonies for 4 common pathogens. 3 foragers were sampled in each colony, which provided 3 technical replicates each (n=9 per colony per virus). Replicates without amplification were excluded. Cq values above ~30 (marked with a horizontal line) are generally considered to indicate no significant infection. ABPV – acute bee paralysis virus. CBPV – chronic bee paralysis virus. DWV – deformed wing virus. SBV – sacbrood virus.

Journal of Experimental Biology • Supplementary information

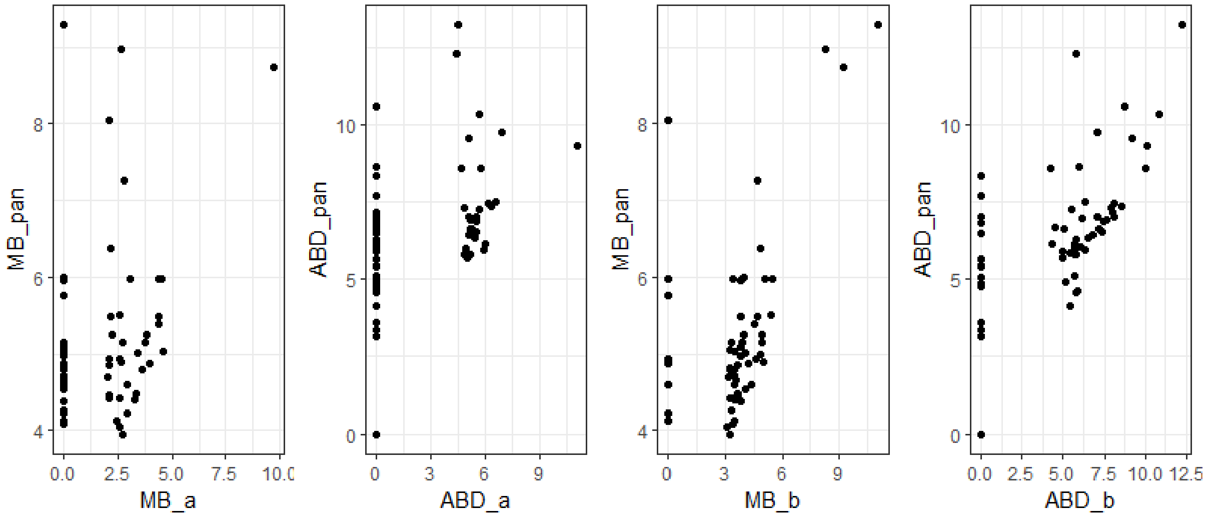

Fig. S2. Scatterplots showing correlation between log_{10} DWV loads in the mushroom bodies (MB) and abdomens (ABD), estimated using different primers sets for DWV-A strain, DWV-B strain and pan-DWV primers (capable to amplify both strains).

Table S1. List of honey bee genome genes implicated in brain GABA signalling.

NCBI gene id	Aliases	Literature evidence & functional annotations
Camkii	Calcium/calmodulin-dependent protein kinase II	Phosphorylates synapsin, likely leading to activation (Sadanandappa et al., 2013)
Gat-1B	GABA neurotransmitter transporter-1B	
Gat-a	GABA neurotransmitter transporter-1A	
Grd	GABA-gated ion channel	Involved in forming ionotropic GABA receptors in *Drosophila* (Gisselmann et al., 2004)
LCCH3	ligand-gated chloride channel homolog 3	Involved in forming ionotropic GABA receptors in *Drosophila* (Gisselmann et al., 2004)
LOC113218647	gamma-aminobutyric acid type B receptor subunit 1	
LOC406124	Rdl; gamma-aminobutyric acid receptor subunit beta	Inhibits learning in *Drosophila* (Liu et al., 2007); ionotropic receptor (Boitard et al. 2015)
LOC408381	Na- and Cl-dependent GABA transporter 1	
LOC408432	Gad1; glutamate decarboxylase	Implicated in GABA shunt (Kanehisa & Goto, 2000); involved in GABAergic signalling into the mushroom body, antagonistic for olfactory learning (Liu & Davis, 2008)
LOC408559	retinal dehydrogenase 1	Annotated to GABA synthesis in KEGG (Kanehisa & Goto, 2000)
LOC408955	Gabat; gamma-aminobutyric acid transaminase	
LOC409065	TBPH; TAR DNA-binding protein-43 homolog	Regulates Gad1 in *Drosophila* (Romano et al., 2021)
LOC409089	VGAT; vesicular inhibitory amino acid transporter	Integral to GABA vesicle transport and GABA synaptic vesicle membrane (Fei et al., 2010)
LOC410140	gamma-aminobutyric acid type B receptor subunit 2	
LOC410979	gamma-aminobutyric acid type B receptor subunit 2	
LOC411140	putative aldehyde dehydrogenase family 7 member A1	Annotated to GABA synthesis in KEGG (Kanehisa & Goto, 2000)
LOC411729	Na- and Cl-dependent GABA transporter	
LOC412177	4-aminobutyrate aminotransferase	Annotated to GABA shunt pathway in KEGG (Kanehisa & Goto, 2000)
LOC412305	succinate-semialdehyde dehydrogenase	Annotated to GABA shunt pathway in KEGG (Kanehisa & Goto, 2000)
LOC413596	receptor-type guanylate cyclase gcy-4	
LOC550687	aldehyde dehydrogenase, mitochondrial	Annotated to GABA synthesis in KEGG (Kanehisa & Goto, 2000)
LOC551737	Syn; Synapsin	CNS GABA release and olfactory learning (Michels et al., 2005; Sadanandappa et al., 2013)
LOC725184	autophagy-related 8a; GABA receptor assoc. protein	Annotated to GABA receptor binding in Gene Ontology (Gaudet et al., 2011)
LOC726514	adenylate cyclase type 6	Critical for PER and gustatory processing, implicated in GABA signalling (Paranjpe et al., 2012)

Table S2. Spearman correlation analyses between DWV-A, DWV-B and pan-DWV primers.

tissue	primer comparison	correlation coefficient	p-value
**MBs**	pan-DWV x DWV-A	0.266	0.040
**MBs**	pan-DWV x DWV-B	0.512	<0.001
**abdomens**	pan-DWV x DWV-A	0.438	<0.001
**abdomens**	pan-DWV x DWV-B	0.585	<0.001

Table S3. Coefficients fit by a generalized linear model to normalised GABA-related gene expression data, and results of coefficients' one-sample T-test for the significant difference from zero.

Variable	Levels	Coefficient			T-value	p-value	sig.
		α estimate	β estimate	StErr			
constant	*Abat*, BR	1.173	-	0.529	2.218	0.028	*
behavioural group	GR	-2.132	-	0.430	-4.962	1.91×10^{-6}	***
gene	*Camkii*	-0.718	-	0.675	-1.063	0.290	ns
	Gad1	-0.669	-	0.675	-0.991	0.323	ns
	Rdl	-0.817	-	0.675	-1.210	0.228	ns
	Syn	-0.725	-	0.675	-1.073	0.285	ns
log_{10} DWV GE	BR	-	-0.248	0.134	-1.849	0.067	ns
log_{10} DWV GE * behavioural group	GR	-	0.472	0.108	4.356	2.38×10^{-5}	***
log_{10} DWV GE * gene	*Camkii*	-	0.321	0.170	1.891	0.061	ns
	Gad1	-	0.300	0.170	1.767	0.079	ns
	Rdl	-	0.343	0.170	2.017	0.046	ns
	Syn	-	0.295	0.170	1.733	0.085	ns

Journal of Experimental Biology • Supplementary information

Supplementary Materials and Methods

Details of selection process for GABA-related genes

The NCBI Database was searched for annotated genes and gene models containing the keywords “GABA” and “gamma-aminobutyric acid”. The search was initially performed for *Apis mellifera* and then for *Drosophila melanogaster* orthologs (Z. Zhang et al., 2000). Next, KEGG (Kyoto Encyclopaedia of Genes and Genomes) and GO (GeneOntology) annotations for *A. mellifera* and *D. melanogaster* were screened for terms like “GABA shunt”, “GABA synthesis”, and “GABA receptor binding” (Kanehisa & Goto, 2000). This protocol resulted in a shortlist of 24 GABA-related genes (Table S1). Five genes were selected for investigation in the lab with RT-qPCR: this was based on evidence from the literature for involvement in PER or learning, and according to evidence from an RNAseq dataset assessing bee brain gene expression in foragers with different associative learning abilities (unpublished data).

Primer design and validation

To design specific primers for the five genes selected, the longest exon was targeted using resources from HymenopteraMine (Walsh et al., 2022). Exon sequences were uploaded into the IDT PrimerQuestTM tool and a range of different options for primer sets was obtained using the default settings for an intercalated dye qPCR. One exception was the GC clamp which was set at 2 nucleotides. Once the tool identified suitable amplicons, the forward and reverse primers were queried in BLAST against *A. mellifera* sequences in the nr/nt nucleotide database (Z. Zhang et al., 2000), to confirm that no primer pair had any non-specific alignments (E-score ≤ 1.0) in the honey bee genome. Efficiencies of primers were tested using a pool of cDNA samples from the same cohort of bees and 10X serial dilutions. Melting curve analyses were examined to confirm the specificity of primers before proceeding with the focal GR and BR samples.

Journal of Experimental Biology • Supplementary information

References

Boitard, C., Devaud, J.-M., Isabel, G., & Giurfa, M. (2015). GABAergic feedback signaling into the calyces of the mushroom bodies enables olfactory reversal learning in honey bees. *Frontiers in Behavioral Neuroscience*, 9(JULY), 198.

Fei, H., Chow, D. M., Chen, A., Romero-Calderón, R., Ong, W. S., Ackerson, L. C., Maidment, N. T., Simpson, J. H., Frye, M. A., & Krantz, D. E. (2010). Mutation of the Drosophila vesicular GABA transporter disrupts visual figure detection. *The Journal of Experimental Biology*, 213(Pt 10), 1717–1730.

Gaudet, P., Livstone, M. S., Lewis, S. E., & Thomas, P. D. (2011). Phylogenetic-based propagation of functional annotations within the Gene Ontology consortium. *Briefings in Bioinformatics*, 12(5), 449–462.

Gisselmann, G., Plonka, J., Pusch, H., & Hatt, H. (2004). Drosophila melanogaster GRD and LCCH3 subunits form heteromultimeric GABA-gated cation channels*. British Journal of Pharmacology*, 142(3), 409–413.

Kanehisa, M., & Goto, S. (2000). KEGG: kyoto encyclopedia of genes and genomes. *Nucleic Acids Research*, 28(1), 27–30.

Liu, X., & Davis, R. L. (2008). The GABAergic anterior paired lateral neuron suppresses and is suppressed by olfactory learning. *Nature Neuroscience* 2008 12:1, 12(1), 53–59.

Liu, X., Krause, W. C., & Davis, R. L. (2007). GABAA Receptor RDL Inhibits Drosophila Olfactory Associative Learning. *Neuron*, 56(6), 1090–1102.

Michels, B., Diegelmann, S., Tanimoto, H., Schwenkert, I., Buchner, E., & Gerber, B. (2005). A role for Synapsin in associative learning: The Drosophila larva as a study case. *Learning & Memory*, 12(3), 224–231.

Paranjpe, P., Rodrigues, V., Raghavan, K. V., & Ramaswami, M. (2012). Gustatory habituation in Drosophila relies on rutabaga (adenylate cyclase)-dependent plasticity of GABAergic inhibitory neurons. *Learning & Memory*, 19(12), 627–635.

Romano, G., Holodkov, N., Klima, R., & Feiguin, F. (2021). TDP-43 regulates GAD1 mRNA splicing and GABA signaling in Drosophila CNS. *Scientific Reports* 2021 11:1, 11(1), 1–8.

Sadanandappa, M. K., Redondo, B. B., Michels, B., Rodrigues, V., Gerber, B., VijayRaghavan, K., Buchner, E., & Ramaswami, M. (2013). Synapsin function in GABA-ergic interneurons is required for short-term olfactory habituation. *The Journal of Neuroscience : The Official Journal of the Society for Neuroscience*, 33(42), 16576–16585.

Journal of Experimental Biology • Supplementary information
